# Comparison of 6 DNA extraction methods for isolation of high yield of high molecular weight DNA suitable for shotgun metagenomics Nanopore sequencing to detect bacteria

**DOI:** 10.1186/s12864-023-09537-5

**Published:** 2023-08-04

**Authors:** Mathieu Gand, Bram Bloemen, Kevin Vanneste, Nancy H. C. Roosens, Sigrid C. J. De Keersmaecker

**Affiliations:** Transversal Activities in Applied Genomics, Sciensano, Rue Juliette Wytsman 14, 1050 Brussels, Belgium

**Keywords:** DNA extraction, Method evaluation, HMW DNA, Nanopore, Metagenomics, Long-read

## Abstract

**Background:**

Oxford Nanopore Technologies (ONT) offers an accessible platform for long-read sequencing, which improves the reconstruction of genomes and helps to resolve complex genomic contexts, especially in the case of metagenome analysis. To take the best advantage of long-read sequencing, DNA extraction methods must be able to isolate pure high molecular weight (HMW) DNA from complex metagenomics samples, without introducing any bias. New methods released on the market, and protocols developed at the research level, were specifically designed for this application and need to be assessed.

**Results:**

In this study, with different bacterial cocktail mixes, analyzed as pure or spiked in a synthetic fecal matrix, we evaluated the performances of 6 DNA extraction methods using various cells lysis and purification techniques, from quick and easy, to more time-consuming and gentle protocols, including a portable method for on-site application. In addition to the comparison of the quality, quantity and purity of the extracted DNA, the performance obtained when doing Nanopore sequencing on a MinION flow cell was also tested. From the obtained results, the Quick-DNA HMW MagBead Kit (Zymo Research) was selected as producing the best yield of pure HMW DNA. Furthermore, this kit allowed an accurate detection, by Nanopore sequencing, of almost all the bacterial species present in a complex mock community.

**Conclusion:**

Amongst the 6 tested methods, the Quick-DNA HMW MagBead Kit (Zymo Research) was considered as the most suitable for Nanopore sequencing and would be recommended for bacterial metagenomics studies using this technology.

**Supplementary Information:**

The online version contains supplementary material available at 10.1186/s12864-023-09537-5.

## Background

One of the remarkable revolutions of the past decades, is the emergence and democratisation of the Next Generation Sequencing (NGS) methods, which consist of sequencing, such as the Whole Genome Sequencing (WGS) of organisms, in high-throughput. The use of NGS increased year after year, until becoming an integrated part of the molecular tools used for pathogenic bacterial characterization from clinical, veterinary, food/feed or environmental samples [1, 2]. Being more accurate and informative than the conventional techniques, WGS on bacterial isolates offers an all-in-one method able to provide data on subtypes and relatedness, as well as on virulence and anti-microbial resistance (AMR) genes composition [3–6]. Going one step further, shotgun metagenomics, known as the sequencing of all genetic material (metagenome) present in a sample, makes the culturing of the microorganisms from the samples unnecessary and avoids the need of a priori knowledge of what pathogen to look for [7–9]. Using appropriate bioinformatics tools, the relative abundance of each bacterial species composing the sample can be estimated [10], these species can be typed at the strain level and their AMR and virulence genes composition can be determined [11–13]. Moreover, metagenomics has even the potential to discover new pathogens and emerging diseases [14]. This opens up interesting opportunities for a fast diagnosis.

Two major sequencing techniques can be discriminated: the widely implemented second generation techniques consisting of the massive parallel sequencing of short-reads (~ 150—300 bp), and the more recent third generation techniques, comprising the sequencing of single molecules in long-reads up to 4 Mb [1, 2, 7]. Accurate short-read sequencing has some limitations when it comes to genome assembly and resolution of genomic regions containing repeated sequences. In comparison, despite a higher (but constantly improving) error rate, long-read sequencing allows a better understanding of genetic contexts, including the location and arrangement of bacterial AMR and virulence genes present in the chromosome or on mobile elements [15, 16]. A commonly used platform for long-read sequencing is the MinION device, a small pocket-size instrument developed by Oxford Nanopore Technologies (ONT), that offers a portable solution for real-time on-site sequencing [17–19].

To take advantage of the full power of long-read sequencing, it is important to be able to isolate high molecular weight (HMW) DNA during the preliminary experimental procedures that are DNA extraction and purification. Most of the available DNA extraction kits designed for bacteria were first developed for PCR-like reactions and short-read sequencing. Although rapid, efficient and adapted to these applications, these kits frequently include standard bead-beating for cell lysis, and centrifugation steps with spin-column for nucleic acids purification, resulting in DNA shearing that potentially affects the length of the purified DNA fragments [20]. Therefore, researchers started to look for alternatives to purify HMW DNA and some gentle old school methods such as DNA purification using phenol–chloroform and gravity column became popular again [20–22]. The Solid-Phase Reversible Immobilization (SPRI) system, consisting of the binding of DNA to magnetic beads during the washing step, is also used for DNA purification and can help to selectively purify long-fragments. For the bacterial cell lysis step, the use of lytic enzymes, such as lysozyme, can be preferred to bead-beating to not damage the DNA. Nevertheless, these gentle alternatives are more time-consuming, requiring more steps and are often using hazardous chemical products, such as phenol–chloroform, which should be preferably avoided because of environmental considerations. Moreover, in view of future development of on-site DNA extraction methods compatible with the portability offered by Nanopore sequencing, these features are rather not adapted to an easy external use outside of the laboratory, by non-expert users and without specific equipment [18, 19]. Finally, when using these gentle DNA extraction procedures for metagenomics application, it is important to assess the proper and equal lysis of all the bacteria present in a sample, and to keep the correct representation of easy (Gram-negative) and hard (Gram-positive) to lyse bacterial cells. A balance has to be found, between efficient cell lysis allied to easy and rapid purification, and gentle isolation of HMW-DNA which can be complex and time-consuming.

Manufacturers started to release new kits on the market, specifically designed for metagenomics using long-read sequencing. The Quick-DNA HMW MagBead kit from Zymo Research is one of those [23]. Therefore, it is interesting to evaluate this kit for long-read sequencing, and to compare its performance with other existing methods. Whereas several studies have compared DNA extraction methods for metagenomics applications using short-read sequencing [21, 24–28], only few have realized the same with long-read sequencing. For those including long-read sequencing, a limited number of DNA extraction kits were included, however not simultaneously comparing various cell lysis and DNA purification technologies such as bead-beating, lysis buffer, lytic enzymes, phenol–chloroform, spin/gravity column and magnetic beads [23, 29, 30]. Additionally, the effect of the sample matrix on the correct recovery of bacterial species belonging to a spiked defined microbial community and present at different concentrations, was not investigated for these different technologies, while the use of a mock community standard is recommended for the development, optimization and comparison of metagenomics methods [31]. In the present study, we compared 6 DNA extraction protocols with the use of defined bacterial communities, pure and spiked into a synthetic fecal matrix. Based on this comparison, we selected the Quick-DNA HMW MagBead kit as being the most suitable of the tested methods for metagenomics studies focusing on bacteria and using Nanopore sequencing.

## Material and methods

### Preparation of the bacterial mixes

**Fig. 1:**
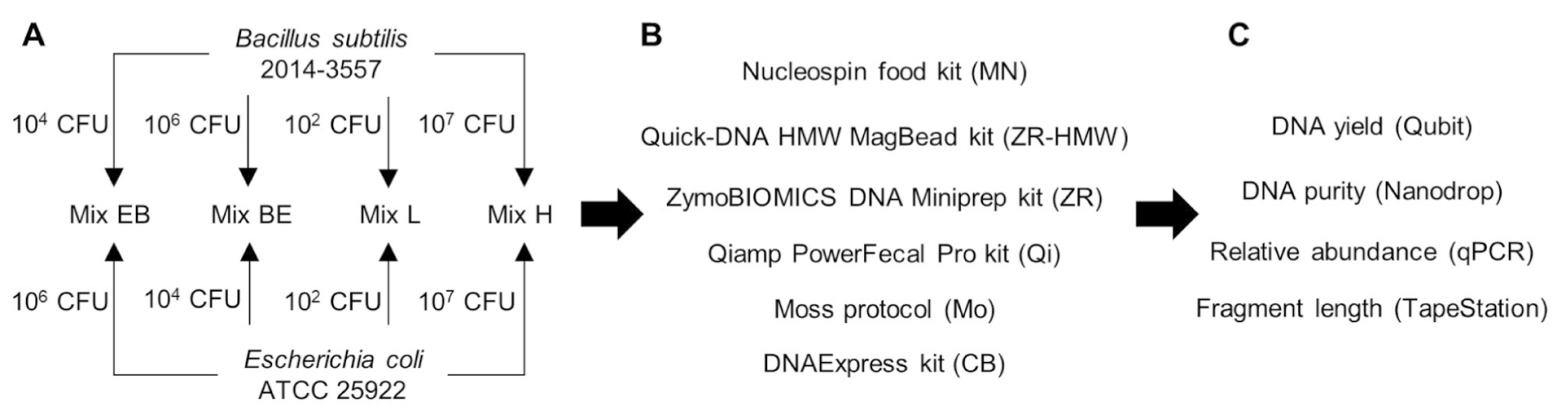
Experimental design for the evaluation of 6 DNA extraction methods using mixes of Gram-positive and Gram-negative bacteria present at various final concentrations. Part A: Composition of four bacterial mixes made of different final cell concentrations of Gram-positive *B. subtilis* and Gram-negative *E. coli*; Mix EB: *E. coli* is overrepresented in comparison with *B. subtilis*; Mix BE: *B. subtilis* is overrepresented in comparison with *E. coli*; Mix L: *B. subtilis* and *E. coli* are mixed at Low concentration; Mix H: *B. subtilis* and *E. coli* are mixed at High concentration; CFU: Colony Forming Units; All mixes were prepared in triplicates. Part B: all mix preparations were processed with 6 DNA extraction methods using different extraction principles (detailed in Table 1); MN: Nucleospin kit; ZR-HMW: Quick-DNA HMW MagBead kit; ZR: ZymoBIOMICS DNA Miniprep kit; Qi: Qiamp PowerFecal Pro kit; Mo: Moss protocol; CB: DNAExpress kit. Part C: the quantity, quality and purity of the extracted DNA were assessed using the instruments listed between brackets

The Gram-positive bacterium *Bacillus subtilis* 2014–3557 [32, 33] and the Gram-negative bacterium *Escherichia coli* ATCC 25922 were cultured in Brain Heart Infusion (BHI) broth (Oxoid, Basingstoke, United-Kingdom) at 30 °C during 48 h and 37 °C during 24 h, respectively. After incubation, a fraction of each culture was taken to determine the cell concentration by plate numbering on Nutrient agar (Oxoid, Basingstoke, United-Kingdom). The remaining part of the cultures was centrifuged at 6000 × *g* for 5 min to pellet the cells, and the culture media was discarded. The cells were resuspended in DNA/RNA shield storage solution (Zymo Research, Irvine, USA) and stored in aliquots at -20 °C until preparation of the mixes for DNA extraction. Four different *B. subtilis* and *E. coli* mixes were prepared with various final cell amounts ranging from 10^2^ to 10^7^ CFU for each species, in final volumes corresponding to the recommended input of tested methods. The composition of the four mixes can be found in Fig. 1. In Mix EB the Gram-negative bacterium *E. coli* was overrepresented (10^6^ CFU) in comparison with the Gram-positive bacterium *B. subtilis* (10^4^ CFU), whereas this was the opposite for Mix BE. In Mix L and Mix H, the 2 bacteria were both present at low (10^2^ CFU) and high (10^7^ CFU) concentration, respectively. For the preparation of the mixes, aliquots of *B. subtilis* and *E. coli* cells stored in DNA/RNA shield were thawed and the storage solution was removed after pelleting the samples by centrifuging 5 min at 6000 × *g*. The samples were then resuspended in Phosphate-Buffered Saline (PBS) (Thermo Fisher Scientific, Waltham, USA), diluted and combined together, to prepare the 4 mixes described in Fig. 1. The mixes were prepared in triplicates, the same day as processed for DNA extraction, in final volumes corresponding to the sample input of each evaluated method, except for Method MN (see section “DNA extraction methods”).

A commercial more complex microbial community, containing 8 bacteria (3 Gram-negative and 5 Gram-positive), was also used in this study: the ZymoBIOMICS Microbial Community Standard II (MCSII) (Zymo Research, Irvine, USA). Although the MCSII contains also 2 fungal strains, those were not investigated in the present study as the compared methods were evaluated for extraction and purification of DNA from bacterial origin. The bacterial species present in this MCSII are mixed in a log-distributed abundance going from 89.1% (*Listeria monocytogenes*) to 0.000089% (*Staphylococcus aureus*) of total genomic DNA (Table 1).

**Table 1:** Bacterial composition of the ZymoBIOMICS Microbial Community Standard II (MCSII)

Species^1^	Gram	GC content (%)	Defined composition^2^ (%)
*Listeria monocytogenes*	+	38.0	89.1
*Pseudomonas aeruginosa*	*-*	66.2	8.9
*Bacillus subtilis*	+	43.9	0.89
*Escherichia coli*	*-*	46.7	0.089
*Salmonella enterica*	*-*	52.2	0.089
*Lactobacillus fermentum*	+	52.4	0.0089
*Enterococcus faecalis*	+	37.5	0.00089
*Staphylococcus aureus*	+	32.9	0.000089

The data presented in this table were communicated by the manufacturer of the MCSII

^1^The MCSII contains also two fungal strains (*Saccharomyces cerevisiae* and *Cryptococcus neoformans*) that were not investigated in this study,

^2^The defined composition represents the percentage of genomic DNA from each species in the MCSII

The MCSII was analyzed 'pure' or 'spiked'. Briefly, 375 µl of the MCSII was centrifuged 5 min at 6000 × *g* and the supernatant was discarded. The cell pellet was subsequently resuspended in 200 µl of PBS (Thermo Fisher Scientific, Waltham, USA) for the 'pure' condition or in 200 µl of Human-based Synthetic Stool Mix (w/ HSA & HgDNA) (Claremont BioSolutions, Upland, USA) for the 'spiked' condition. The synthetic stool matrix is a mixture mimicking stool and the inhibitors commonly found in real human stool samples. This product is described by the manufacturer as a collection of key possible PCR-inhibitory compounds, at relevant amounts in actual stool, based on literature (i.e., bile salts, mucin, serum albumin, dextran sulfate, eukaryotic DNA, etc.…). The main advantage of this synthetic product is avoiding the ethical issues linked to the use of human samples for sequencing experiments.

### DNA extraction methods

Six DNA extraction methods, abbreviated MN, ZR-HMW, ZR, Qi, Mo and CB (Table 2), were evaluated in this study. Five were commercial kits and one (Mo) was published by Moss et al. in 2020 [20]. The methods are using different combinations of lysis and purification methods (Table 2). Cell lysis was achieved with the use of lysis buffers (chemical), lytic enzymes (enzymatic digestion) or bead-beating.

**Table 2:** List of the evaluated DNA extraction methods

Method	Name	Source	Lysis method	Purification method	Portable^1^	Application^2^
MN	Nucleospin food kit	Macherey Nagel	Chemical	Spin-column	No	DNA extraction from food, plant and bacteria. Resulting DNA can be used with various detection methods, especially PCR-like technologies Can be used for Genetically Modified Organisms detection
ZR-HMW	Quick-DNA HMW MagBead kit	Zymo Research	Enzymatic digestion	Magnetic beads	No	Purification of HMW DNA from various samples (including biological fluids, cells, solid tissues and environmental samples) to use in sensitive downstream applications including long read (Oxford Nanopore™) and NGS sequencing, qPCR and arrays
ZR	ZymoBIOMICS DNA Miniprep kit	Zymo Research	Bead beating + chemical	Spin column	No	Purification of DNA from a wide array of sample inputs (e.g. feces, soil, water, biofilms, etc.), that is immediately ready for microbiome or metagenome analyses
Qi	Qiamp PowerFecal Pro kit	Qiagen	Bead beating + chemical	Spin column	No	Isolation of microbial and host genomic DNA from stool and gut samples for PCR, qPCR and NGS
Mo	Moss protocol	[20]	Enzymatic digestion + phenol–chloroform	Gravity column	No	Isolation of high molecular weight DNA from stool or cultured bacteria, suitable for long-read sequencing
CB	DNAexpress kit	Claremont Bio	Bead beating + chemical	Syringe column	Yes	Rapid and easy isolation of high molecular weight DNA from easy to hard to lyse cells present in various complex matrices (including stool, sputum, blood, soil, and tissue) for NGS

^1^The kit can be used as such without specific laboratory equipment except basic consumables such as microtubes, micropipettes and tips

^2^According to the manufacturer or author

In one case, the enzymatic digestion was followed by extraction using a phenol–chloroform solution. After cell lysis, subsequent purification was mostly performed with the use of DNA binding columns, using centrifugation force, a syringe system or simply by gravity flow. Another purification system consisted of the use of DNA binding magnetic beads. Among the 6 methods, method CB claimed to be portable, meaning that no particular laboratory equipment, except micropipettes, tips and microtubes, is needed for its use **(**Table 2**)**. The four *B. subtilis* and *E. coli* bacterial mixes (Mix EB, Mix BE, Mix L and Mix H), prepared in triplicates, were all processed with the six DNA extraction methods. All the DNA extractions were performed according to the manufacturer/author's instructions, with minor adaptations for sample input. For method MN, the recommended sample input is 200 mg of solid sample, which was not feasible with the bacterial mixes. Therefore, the final dilution, made during the preparation of the bacterial mixes to achieve the target final concentrations of each mix, was performed directly in 550 µL of buffer CF, not exceeding a total volume of 565 µL to minimize the impact. Proteinase K (supplied with the kit) was added according to the protocol of the kit and the sample was further process for incubation with no other modifications. For method ZR-HMW, the procedure "Enzymatic Digestion of Microbes" was followed, stepping directly to "Microbial Lysis" as the samples were stored in DNA/RNA shield. For the enzymatic digestion, a solution of lysozyme from egg white (Roche, Bâle, Switzerland) at 100 mg/ml was prepared. For method Mo, the final dilution of the bacterial mixes was performed in 500 µl and this was used as input for the Moss protocol. For Methods ZR, Qi and CB, 250 µl of the bacterial mix was added to the tubes containing the beads and the protocols ("DNA extraction protocol for cells" section for Method CB) were followed without modifications.

A volume of 200 µl of the MCSII 'pure' and 'spiked' samples were processed with methods ZR-HMW, Qi and Mo with the same protocol as for *B. subtilis* and *E. coli* bacterial mixes, except that for Method ZR-HMW, 10 µl of the MetaPolyzyme solution prepared for Method Mo was used instead of lysozyme during Microbial Lysis. Additionally, 200 µl of PBS (PBS 'unspiked') and Synthetic Stool Mix (Stool 'unspiked') were also processed with the same methods, serving as negative controls.

### DNA quantity and quality measurement

The extracted DNA yield was determined using the Invitrogen Qubit 4 Fluorometer (Thermo Fisher Scientific, Waltham, USA) with Qubit dsDNA HS Assay Kit (Thermo Fisher Scientific, Waltham, USA), having a range of detection between 0.05 and 120 ng/µl for an analysis volume of 2 µl. The purity of the DNA extracts was evaluated with their absorbance measured at 230 nm, 260 nm and 280 nm with the NanoDrop 2000 Spectrophotometer (Thermo Fisher Scientific, Waltham, USA). According to the manufacturer’s specifications, pure DNA has a 260/280 ratio around 1.8, and a 260/230 ratio comprised between 2.0 and 2.2. The average fragment length of the extracted DNA was determined by using the 4200 TapeStation System (Agilent, Santa Clara, USA) with the Genomic DNA ScreenTape and reagents (Agilent, Santa Clara, USA). Finally, the relative abundance of *B. subtilis* and *E. coli* DNA, extracted from the bacterial mixes, was estimated by qPCR. For the detection of *B. subtilis*, the VitB2-UGM qPCR TaqMan assay from Barbau-Piednoir et al. [32] was used with following modification: the reaction was performed in 1X Takyon ROX MasterMix UNG (Eurogentec, Liège, Belgium). A positive control, included in each vitB2-UGM qPCR assay, was prepared by extracting the DNA from a 48 h *B. subtilis* 2014–3557 culture in BHI (Oxoid, Basingstoke, United-Kingdom) at 30 °C, with the GenElute Bacterial Genomic DNA Kit (Sigma-Aldrich, Saint-Louis, USA) following the procedure for Gram-positive bacteria, including a cell lysis step with lysozyme from egg white (Roche, Bâle, Switzerland). For the detection of *E. coli*, the SYBR green qPCR assay from Barbau-Piednoir et al. [34] with *uidA*-3 primer pair was used. A positive control, included in each *uidA*-3 qPCR assay, was prepared by extracting the DNA from a 24 h *E. coli* ATCC 25922 culture in BHI (Oxoid, Basingstoke, United-Kingdom) at 37 °C, with the GenElute™ Bacterial Genomic DNA Kit (Sigma-Aldrich, Saint-Louis, USA) following the procedure for Gram-negative bacteria. All qPCR assays were performed on a CFX96 Touch Real-Time PCR Detection System (Bio-Rad, Hercules, USA) and Cq values were automatically determined by the BioRad CFX Maestro 2.0 software (Bio-Rad, Hercules, USA).

To see if the DNA extraction methods had significant effects on Qubit, Nandodrop, TapeStation and qPCR values, statistical analysis was performed on the data using R version 4.1.2 and the rstatix package version 0.7.1. The Kruskal–Wallis test, a non-parametric test adapted for comparison of three or more groups, was used with α value equal to 0.05. For every tested dataset, the null hypothesis, i.e. no influence from the DNA extraction methods, was rejected if the *p*-value was below the alpha value.

### Long-read sequencing

Library preparation was performed using the Ligation sequencing kit for genomic DNA (SQK-LSK109) (Oxford Nanopore Technologies, Oxford, UK). Oxford Nanopore Technologies (ONT) recommends to use 1 µg (in 48 µl) of pure sample as input for library preparation. MCSII 'pure', MCSII 'spiked' and stool 'unspiked' DNA samples selected for Nanopore sequencing from Method ZR-HMW and CB were diluted to 20.8 ng/µl in DNA/RNA nuclease free distilled water (Thermo Fisher Scientific, Waltham, USA) to respect this specification. As the concentration of MCSII 'pure' DNA sample obtained from Method CB was not concentrated enough, the maximum input volume (48 µl) was used for library preparation. The generated libraries were then loaded on a Spot-ON MinION flow cell (FLO-MIN 106D, R9.4.1 version) (Oxford Nanopore Technologies, Oxford, UK) in singleplex and sequencing was performed on a Mk1C device (Oxford Nanopore Technologies, Oxford, UK) for 72 h with live-basecalling off.

### Bioinformatics analysis of the long-read sequencing data

Raw sequencing data were basecalled using Guppy version 5.0.7 (Oxford Nanopore Technologies, Oxford, UK) on a GPU server in super high accuracy mode with config file dna_r9.4.1_450bps_sup.cfg, trim strategy set to DNA and qscore filtering disabled. The basecalled reads were subsequently filtered with NanoFilt version 2.8.0 [35] with minimum quality score and length equal to 7 and 300 bp, respectively, and are available in the NCBI Sequence Read Archive (SRA) repository, under the BioProject ID PRJNA954551. Statistics of the filtered high quality reads were obtained with NanoPlot version 1.36.2 [35].

Similarly as done by Nicholls et al. [31], the relative abundance of the bacterial species present in the MCSII samples ('pure' and 'spiked') was estimated using a mapping-based method. In this study the KMA (*k*-mer alignment) mapper version 1.4.4 was used. Using kma_index, an in-house database was indexed from the full genome sequences of the MCSII species (https://s3.amazonaws.com/zymo-files/BioPool/ZymoBIOMICS.STD.refseq.v2.zip; downloaded in March 2022) and used with KMA. The filtered sequencing data were used as input in KMA with the following settings optimized for long-read data: -mem_mode, -bc set to 0.7, -bcNano, -ef, -proxi set to 0.9, -1t1 and -ca. Additionally, to limit the number of erroneous mapping, the -ID and -mrs options were set to 0.1 and 0.01, respectively, and only results with depth ≥ 0.04 were kept. The relative abundance of each MCSII bacterial species in a sample was estimated by dividing the number of bases that mapped to sequences belonging to a species by the total number of bases determined with NanoPlot. The obtained results were expressed in percent.

To characterize the nature of the background DNA, taxonomic classification was performed using Kraken2 [36, 37] version 2.0.7 with default parameters and an in-house database composed of sequences downloaded from the NCBI RefSeq database on the February 11^th^, 2021. These sequences included all 'Complete Genome' sequences with the accession prefixes NC, NW, AC, NG, NT, NS, and NZ for archaea, bacteria, fungi, protozoa and viruses. Additionally, the database contained also reference sequences for the following birds, mammals, and arthropods: *Anas platyrhynchos* (GCF_015476345), *Columba livia* (GCF_000337935), *Gallus gallus* (GCF_000002315), *Meleagris gallopavo* (GCF_000146605), *Numida meleagris* (GCF_002078875), *Bos taurus* (GCF_002263795), *Capra hircus* (GCF_001704415), *Cavia porcellus* (GCF_000151735), *Chlorocebus sabaeus* (GCF_015252025), *Equus caballus* (GCF_002863925), *Homo sapiens* (GCF_000001405), *Mesocricetus auratus* (GCF_000349665), *Mus musculus* (GCF_000001635), *Rattus norvegicus* (GCF_015227675), *Ovis aries* (GCF_002742125), *Sus scrofa* (GCF_000003025), *Ades aegypti* (GCF_002204515*), Aedes albopictus* (GCF_006496715), *Apis mellifera* (GCF_003254395), *Culex quinquefasciatus* (GCF_015732765), *Ixodes scapularis* (GCF_002892825), and *Stomoxys calcitrans* (GCF_001015335). From the Kraken2 output, all the taxonomic results for which less than 2% of the reads were attributed to a species identification, were considered low confidence, requiring further confirmation by downstream analysis, as Kraken2 is known to produce false positive results when low abundant taxa are reported [38, 39]. With this threshold, the precision was deliberately preferred over the recall, to investigate the origin of the background DNA present in the synthetic stool mix.

### Results

Six DNA extraction methods based on various technologies for cell lysis and DNA purification were compared in this study. To evaluate these methods, four bacterial mixes composed of different concentrations of the Gram-positive *B.* *subtilis* and the Gram-negative *E. coli* were used. The DNA yield, purity and length as well as the relative abundance of the two species were assessed in each DNA extract. From this first comparison, the three best kits were selected for further evaluation using a commercial microbial community standard (MCSII) containing 8 bacterial strains in log distribution, pure in PBS (MCSII 'pure') or spiked in a synthetic fecal matrix (MCSII 'spiked'). The PBS and synthetic fecal matrix were also processed alone, without spiking (PBS 'unspiked' and stool 'unspiked'). After similar DNA quantity and quality evaluation as performed during the first comparison, samples from two kits were selected for long-read sequencing using the Nanopore platform.

### Performance evaluation of the methods for DNA yield, purity and fragment length using mixes of *B. subtilis* and *E. coli*

Four *B. subtilis* and *E. coli* bacterial mixes (Mix EB, Mix BE, Mix L and Mix H; Fig. 1) were processed with the six selected DNA extraction methods (MN, ZR-HMW, ZR, Qi, Mo, and CB; Table 2). The obtained DNA extracts were quantified and their purity and fragment length were assessed. Only results of the Mix H are shown in Table 3, as for the other mixes, most of the values were out of range because they were below the limit of the detection of Qubit, Nanodrop and/or TapeStation (Supplementary Material 1).

**Table 3:** DNA quantity, purity and fragment length obtained after extraction of *B. subtilis* and *E. coli* mix H using the evaluated DNA extraction methods

Method	Elution volume^1^(µl)	Concentration^2†^(ng/µl)	Yield^3^(ng)	A260/280^4^	A260/230^4^	Average size^5†^ (bp)
MN	100	0.84 ± 0.06	84.1 ± 5.5	Out of range	Out of range	36,445 ± 17,282
ZR-HMW	50	7.47 ± 0.70	373.3 ± 34.9	2.0 ± 0.1	1.5 ± 0.1	31,220 ± 691
ZR	100	0.36 ± 0.25	35.9 ± 24.8	Out of range	Out of range	Out of range
Qi	60	2.88 ± 0.83	172.6 ± 49.6	2.1 ± 0.1	0.1 ± 0.1	15,283 ± 325
Mo	100	0.39 ± 0.27	38.5 ± 26.7	Out of range	Out of range	Out of range
CB	100	2.34 ± 0.49	234.3 ± 49.1	1.9 ± 0.1	1.6 ± 0.0	15,550 ± 187

^1^According to manufacturer/author’s instructions

^2^Average and standard deviation, from triplicate measures determined using Qubit Fluorometer

^3^Average and standard deviation, from triplicate concentration measures multiplied by corresponding elution volumes

^4^Average and standard deviation, from triplicate measures determined using Nanodrop

^5^Average and standard deviation, from triplicate measures determined using TapeStation

^†^DNA extraction methods had significant influence on DNA concentration and average size (Kruskal–Wallis; p < 0.05)

Out of range: the related parameter cannot be measured because the sample concentration is below the limit of detection of Nanodrop (2 ng/µL) or TapeStation (0.5 ng/µL), according to the manufacturer’s specifications

The DNA concentration (n: 18; *p*-value: 0.008) and average fragment size (n: 12; *p*-value: 0.03) differed significantly between the evaluated DNA extraction methods, but not the A260/280 (n: 9; *p*-value: 0.08) and A260/230 (n: 9; *p*: 0.06) Nanodrop ratios (Kruskal–Wallis, α: 0.05). Methods MN, ZR and Mo were not able to generate a concentration of DNA higher than 1 ng/µl. Because of this, purity assessment using Nanodrop was not possible for these 3 methods and average fragment length estimation was possible only for Method MN. The latter obtained the highest value for fragment length (36 445 bp), for all methods considered, although it can be noticed that the triplicate values had a large variability, ranging from 21,305 bp to 55,273 bp (SD ± 17,282), suggesting a lack of repeatability. The remaining 3 methods ZR-HMW, Qi and CB generated higher concentrations of DNA, with Method ZR-HMW being the best (7.47 ± 0.70 ng/µl). In terms of purity for these three methods, A260/280 ratio values were comprised between 1.9 ± 0.1 and 2.1 ± 0.1, which is close to the 1.8 value recommended by the manufacturer for pure sample. For A260/230, none of the 3 methods were in the recommended range of 2.0—2.2, with Method Qi obtaining a particularly low ratio (0.1 ± 0.1), in comparison with Methods ZR-HMW and CB. Finally, between these 3 methods, Method ZR-HMW generated around two times longer fragments (31,220 ± 691 bp) in comparison with Method Qi (15,283 ± 325 bp) and Method CB (15,550 ± 187 bp). Based on this first evaluation, Method ZR-HMW seemed to outperform the other methods. Method Qi and CB performed also well but both with lower fragment length and also with lower purity for Method Qi.

### Relative abundance comparison of *B. subtilis* and *E. coli* in the four bacterial mixes using qPCR

**Fig. 2:**
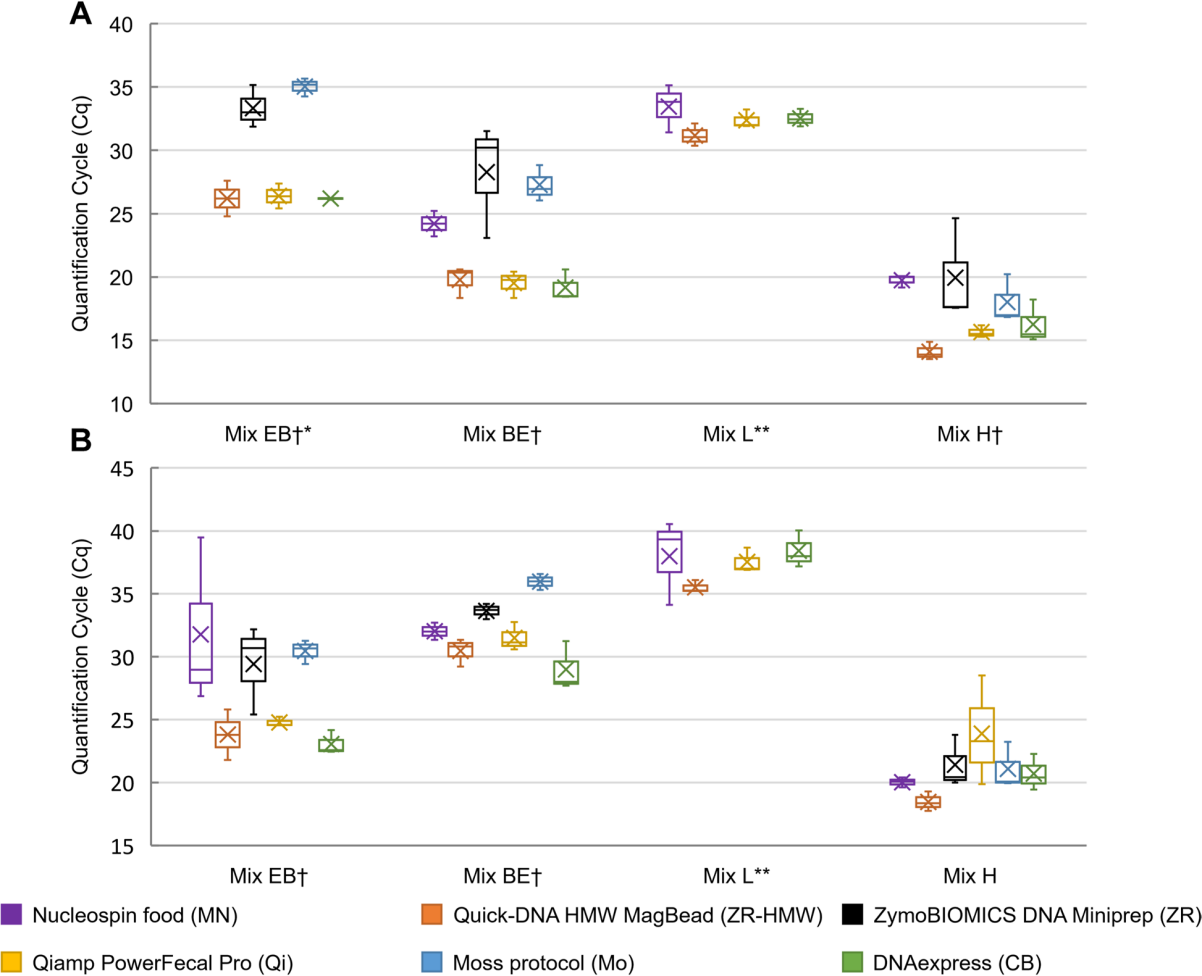
Relative abundance estimation of *B. subtilis* and *E. coli* in the four bacterial mixes after DNA extraction using the evaluated methods. The graphs show quantification cycles (Cqs) obtained by qPCR for the detection of the vitamin B2 producing gene in *B. subtilis* (part A) and the *uidA* gene in *E. coli* (part B). In boxplots the mean and median of triplicate series are represented by a cross and dash, respectively. Mix EB: *E. coli* is overrepresented in comparison with *B. subtilis*; Mix BE: *B. subtilis* is overrepresented in comparison with *E. coli*; Mix L: *B. subtilis* and *E. coli* are mixed at Low quantity; Mix H: *B. subtilis* and *E. coli* are mixed at High quantity. *: *B. subtilis* was not detected when Mix EB samples were processed with Method MN. **: neither *B. subtilis* nor *E. coli* were detected in Mix L when processed with methods ZR and Mo. †: DNA extraction methods had significant influence on detection of *B. subtilis* or *E. coli* (Kruskal–Wallis; *p* < 0.05; Supplementary Material 2)

To have a better estimation of the proportion of extracted DNA belonging to *E. coli* (Gram-negative) and *B. subtilis* (Gram-positive) in the four mixes, qPCR analyses targeting respectively the *uidA* and the vitamin B2 producing genes, were performed on all the triplicate DNA samples obtained from the 6 evaluated methods. The relative abundance of the detected bacteria was estimated with the quantification cycle (Cq) results, i.e. lower Cqs were associated with high abundance and vice-versa (Fig. 2**)**. Details of statistical analysis to evaluate the significant influence of the DNA extraction methods on detection of *B. subtilis* and *E. coli* are available in Supplementary Material 2.

The DNA extraction methods had significant influence on the detection of *B. subtilis* in mix EB (n: 15; *p*-value: 0.03) and mix BE (n: 18; *p*-value: 0.03) DNA extracts. The same can be observed for detection of *E. coli* in mix EB (n: 18; *p*-value: 0.02) and mix BE (n: 18; *p*-value: 0.02) DNA extracts. For Mix EB extracts, where *E. coli* was overrepresented (10^6^ CFU) in comparison to *B. subtilis* (10^4^ CFU), lower Cqs were obtained for the detection of *B. subtilis* and *E. coli*, when processed with Methods ZR-HMW, Qi and CB, in comparison to Methods MN, ZR and Mo (Fig. 2). The same observation can be made for Mix BE extracts, where *B. subtilis* (10^4^ CFU) was overrepresented (10^6^ CFU) in comparison with *E. coli* (10^4^ CFU). When it came to the detection of *B. subtilis* and *E. coli* at low quantity (10^2^ CFU) in Mix L, none of the two species could be retrieved in DNA extracts obtained with methods ZR and Mo, and no significant difference could be observed amongst the other methods (Supplementary Material 2**)**. *B. subtilis* and *E. coli* were better detected from Mix H extracts, containing high quantities of the two species, when processed with Method ZR-HMW, although significant differences amongst the method could only be observed for *B. subtilis* detection (n:18; *p*-value: 0.03). Overall, Method ZR-HMW, Qi and CB were the ones that resulted in the lowest Cqs, while Method MN and Mo had sometimes difficulties to detect the 2 species when present at low quantity.

### Evaluation for Nanopore sequencing

**Fig. 3:**
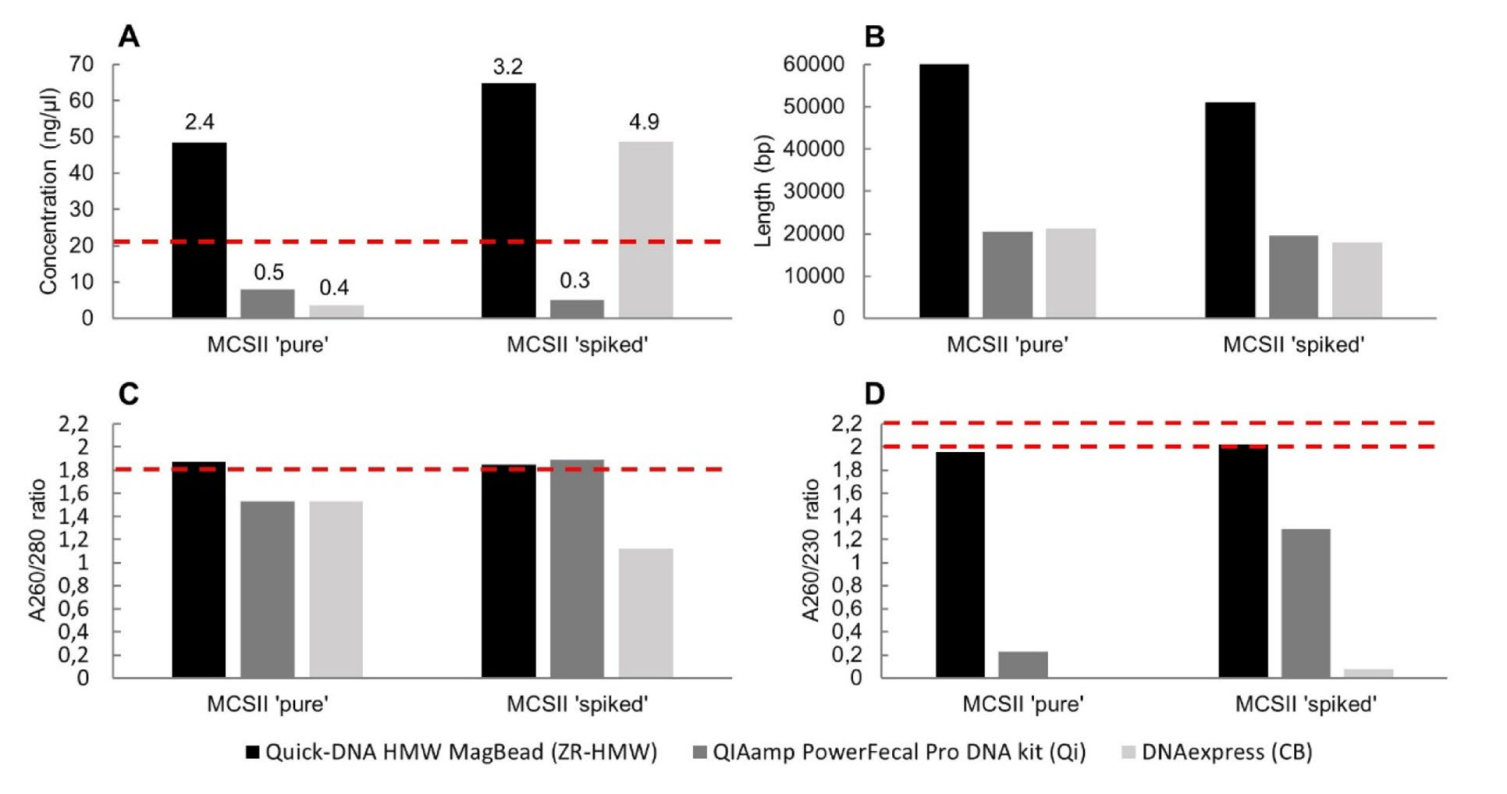
DNA concentration, purity and length of the MCSII samples measured after DNA extraction with Methods ZR-HMW, Qi and CB. MCSII 'pure': MCSII processed pure in PBS. MCSII 'spiked': MCSII spiked in synthetic stool mix. Part A represents DNA concentrations (with corresponding yield in µg at the top of bars) measured with Qubit and the red dotted line indicates the minimum concentration (20.8 ng/µl) required for Nanopore library preparation with the ligation kit. Part B represents average fragment length obtained with TapeStation. Part C represents A260/280 ratio obtained from Nanodrop measurements and the red dotted line represents the ideal minimum value (> 1.8) for pure sample. Part D represents A260/230 ratio obtained from Nanodrop measurement and the red dotted lines represent the interval values (2.0—2.2) for which a DNA sample is considered as pure

Considering the performances of Method ZR-HMW, Qi and CB obtained so far with *B. subtilis* and *E.* *coli* bacterial mixes, these 3 kits were selected for further experiments. The 3 methods were used to extract the DNA from the MCSII samples 'pure' and 'spiked'. As negative controls, PBS 'unspiked' and stool 'unspiked' samples were also processed identically. The DNA yield and quality of the samples was again assessed, similarly as for the *B. subtilis* and *E. coli* bacterial mixes (Fig. 3**)**.

Method ZR-HMW produced a high concentration of pure HMW DNA, except for MCSII 'pure' sample that did not reach the A260/230 range of 2.0—2.2, but was very close (1.96). Concerning samples processed with Method CB, the MCSII 'spike' sample reached the minimum DNA concentration (> 20.8 ng/µl) required for library preparation, but not the MCSII 'pure' sample. The Nanodrop ideal ratios were not reached for these two samples processed with Method CB, with A260/230 values being particularly low. Between two and three times shorter fragments were generated with Method CB in comparison with Method ZR-HMW. For samples processed with these two methods (ZR-HMW and CB), more DNA was produced when the MCSII was spiked in the synthetic stool mix than when processed pure in PBS. This can be explained by the presence of background DNA, as measured in the synthetic stool mix processed alone (stool 'unspiked') and giving a concentration of 42.4 ng/µl and 41 ng/µl with Method ZR-HMW and CB, respectively (Supplementary material 3). For Method Qi samples, despite achieving similar or better purity and fragment length, in comparison with samples from Method CB, none of them reached the minimum concentration of 20.8 ng/µl required for library preparation. Therefore, Method Qi samples were not kept for Nanopore sequencing. On the contrary, MCSII samples processed with Method ZR-HMW fulfilled all the conditions and were further processed for library preparation. Although MCSII samples processed with Method CB did not meet all the required criteria, they were still included for Nanopore sequencing, for comparison purposes with Method ZR-HMW, i.e. laboratory method (ZR-HMW) vs. portable method (CB). Additionally, stool 'unspiked' samples from these two methods (ZR-HMW and CB) were also included for Nanopore sequencing, as they reach the minimum concentration for library preparation, but not the PBS 'unspiked' samples as no DNA could be quantified with the Qubit instrument (Supplementary material 3).

After Nanopore sequencing of the generated libraries, statistics of the high-quality reads were generated (Table 4**)**. For a same sample processed with the two evaluated methods, more data, i.e. a higher number of reads and bases, were generated with Method ZR-HMW in comparison with Method CB. Moreover, the N50 was also higher for Method ZR-HMW, showing that longer DNA fragments were sequenced. Similarly for the two methods, a drop of the number of bases and N50 value is observed between the MCSII processed pure, or spiked into synthetic stool mix. It can be noticed that for each method considered separately, a comparable number of reads was obtained between MCSII samples and stool 'unspiked'. However, the reads were shorter for the stool 'unspiked' sample, and a lower number of bases was generated.

**Table 4:** Statistics of the Nanopore sequencing experiments

Sample	MCSII 'pure'		MCSII 'spiked'		Stool 'unspiked'	
DNA extraction method	ZR-HMW	CB	ZR-HMW	CB	ZR-HMW	CB
Number of reads (K)	1154.9	183.9	1102.4	159.6	1154.4	78.0
Read length N50 (Kb)	21.8	12.0	11.9	0.52	0.52	0.43
Median read quality	13.2	12.1	14.2	14.4	13.2	12.1
Total bases (Gb)	18.2	1.6	1.9	0.094	0.62	0.038

Statistics were obtained with NanoPlot after filtering of the raw sequencing data with NanoFilt. MCSII 'pure': MCSII processed pure in PBS. MCSII 'spiked': MCSII spiked in synthetic stool mix. Stool 'unspiked': the synthetic stool mix processed alone. ZR-HMW: Quick-DNA HMW MagBead kit. CB: DNAexpress kit

### Taxonomic classification and relative abundance estimation of the species present in the sequenced samples

**Fig. 4:**
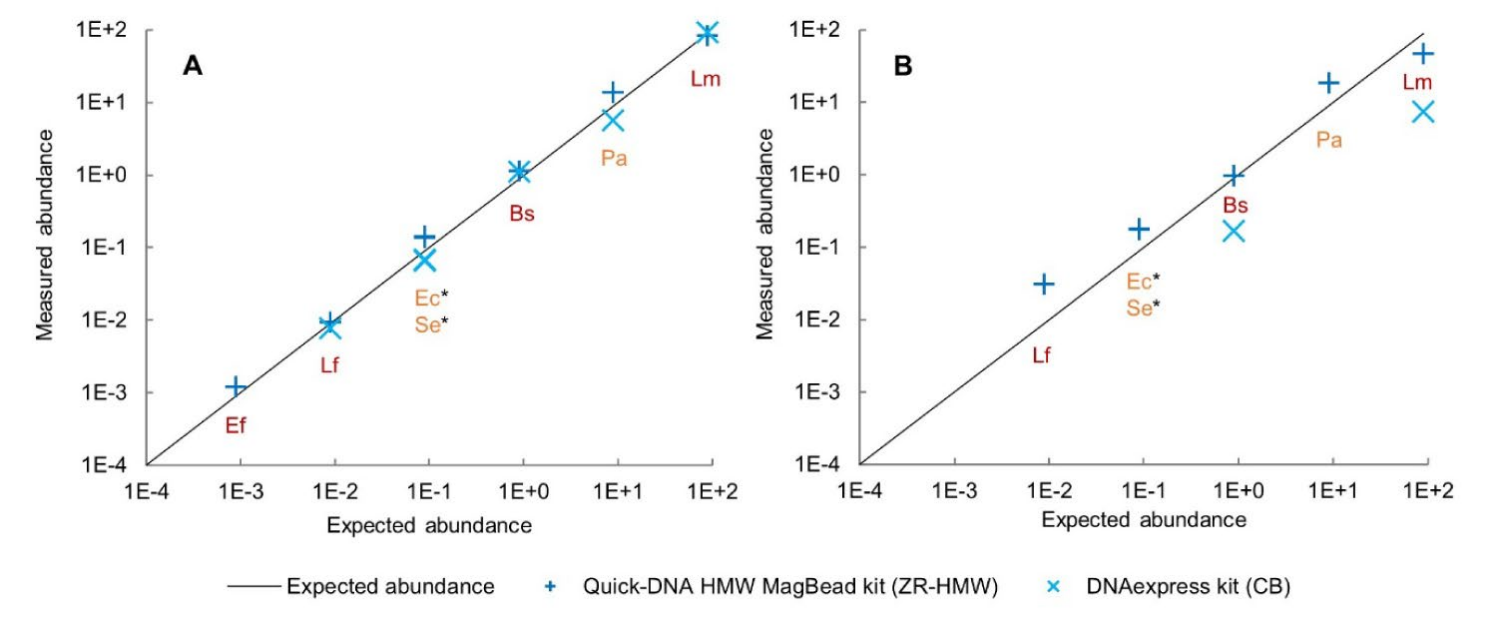
Expected vs. measured abundance of the MCSII bacterial species in the sequenced samples. Percentage of bases that were mapped by KMA to reference sequences of the MCSII bacterial species (y-axis), against the expected percentage of species present in the MCSII (x-axis), in log scale, for *L. monocytogenes* (Lm), *P. aeruginosa* (Pa), *B. subtilis* (Bs), *S. enterica* (Se), *E. coli* (Ec), *L. fermentum* (Lf) and *E. faecalis* (Ef). Gram-negative bacteria are shown in orange and Gram-positive bacteria in red. Part A: MCSII 'pure' sample. Part B: MCSII 'spiked' sample. *: results for *S. enterica* and *E. coli* are overlapping for both methods

The relative abundance of the bacteria present in the MCSII 'pure' and 'spiked' samples was estimated and compared to the expected proportion of genomic DNA (Fig. 4).

When the MCSII was analyzed 'pure' in PBS, the proportions of spiked species (Table 1) were quite correctly respected for the two methods ZR-HMW and CB. It can still be noted that a few species, i.e. *P. aeruginosa*, *E. coli* and *S.* *enterica*, were slightly overestimated by Method ZR-HMW while they were underestimated for Method CB. Additionally, *S. aureus* (the lowest abundant species, 0.000089%) could not be detected in MCSII 'pure' sample, independently of the method used, and *E. faecalis* (the second lowest abundant species, 0.00089%) could only be detected with Method ZR-HMW. When the MCSII was spiked in synthetic stool mix, only *L. monocytogenes* and *B. subtilis* could be detected in Method CB DNA extracts, at a lower percentage than expected. For MCSII 'spiked' samples processed with Method ZR-HMW, the two lowest abundant species *S. aureus* and *E. faecalis* were not detected. Concerning the remaining species, they were detected but *P. aeruginosa*, *E.* *coli*, *S. enterica* and *L.* *fermentum* were overrepresented while *L. monocytogenes* was underrepresented, and *B. subtilis* was detected at the correct proportion.

**Fig. 5:**
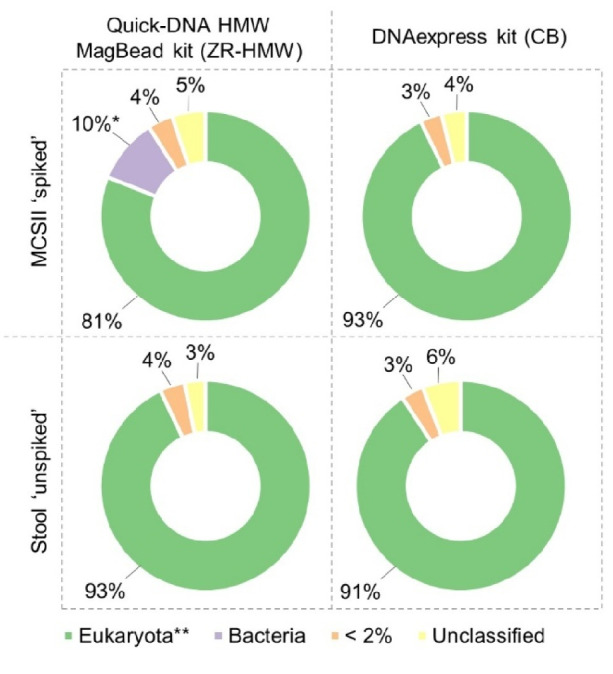
Identification of the background DNA present in the samples including the synthetic stool mix processed with methods ZR-HMW and CB. The graphs show the proportions of reads that were attributed to eukaryota or bacteria using Kraken2. The ‘ < 2%’ category contains all the species identifications for which less than 2% of the reads were attributed. This category is composed of MCSII low abundant species and false positive results (Supplementary material 4). The unclassified category contains all the reads that could not be identified by Kraken2. MCSII 'spiked': MCSII spiked in synthetic stool mix. Stool 'unspiked': synthetic stool mix without MCSII. ZR-HMW: Quick-DNA HMW MagBead kit. CB: DNAexpress kit. *: reads were attributed to *L. monocytogenes* and *P. aeruginosa*; **: all the eukaryote reads were identified as belonging to the species *Sus scrofa* (wild boar)

As a high amount of DNA was previously quantified for the stool 'unspiked' sample (Supplementary material 3), and a high number of short reads were sequenced in Nanopore (Table 4), there was a need to check to what organisms belong these sequences present in the synthetic stool mix. Therefore, taxonomic identification was performed with Kraken2 on sequencing data from stool 'unspiked' samples, as well as from MCSII 'spiked' samples, processed with both Method ZR-HMW and CB. For all samples including the synthetic stool mix, spiked or not with the MCSII, the major part of the reads (> 81%) were attributed to the species *Sus scrofa* independently of the used method (Fig. 5). In the MCSII 'spiked' sample processed with Method ZR-HMW, some bacterial reads (10%) were found and they were attributed to the 2 most abundant species from the MCSII, i.e. *L.* *monocytogenes* and *P.* *aeruginosa*. Independently of the samples and DNA extraction kits, some reads from the category with less than 2% of reads attributed to a species were classified as belonging to MCSII species but they were mixed with false positive results (Supplementary material 4).

## Discussion

To exploit the full power of long-read sequencing, the isolation of HMW DNA is a key parameter. Moreover, in the context of metagenomics studies, equal lysis of the bacteria present in a sample, even at low concentrations, is important to obtain the most accurate representation of its bacterial microbiome. The isolation of pure DNA, free of compounds that can impact downstream analysis, is also mandatory. Considering these parameters, Method ZR-HMW obtained the best results amongst the 6 DNA extraction methods tested in the present study, by generating high yields of pure long-fragment DNA. Additionally, unbiased lysis of Gram-positive and Gram-negative bacteria, present in simple (*B. subtilis* and *E. coli*) and complex (MCSII) bacterial mixes, was obtained with this method. A similar result was obtained by the team of Nicholls et al. who also sequenced the MCSII pure (extracted with different experimental settings) on one flow cell (FLO-MIN106C vs. FLO-MIN106D used in the present study) with refueling, and obtained a comparable yield (16.51 Gb) [31]. Nevertheless, they could detect the least abundant species of the microbial standard, i.e. *S. aureus* (with no more than 4 reads), which was not possible in our case. This difference in terms of limit of detection can most probably be explained by the different bioinformatics workflow used in their study for the interpretation of the data. Indeed, when analyzing their sequencing data (ERR3152366) with KMA and the same settings and threshold as used in the present study, *S. aureus* could not be detected also (data not shown). Sequencing statistics showed that both the ZR-HMW and CB methods were affected by the presence of synthetic stool mix, but this was particularly dramatic for Method CB. As a consequence, Method CB was less performant for retrieving the correct proportions of the MCSII species. The enzymatic digestion combined with purification with magnetic beads was more efficient than bead-beating and purification columns, for the isolation of bacterial HMW-DNA from the MCSII species, in the presence of high concentrations of eukaryotic DNA. Therefore, considering all these results and the different technologies tested in this study for DNA isolation, the combination of enzymatic digestion for lysis and magnetic beads for purification, as used in Method ZR-HMW, appears to be the most suitable for metagenomics studies using Nanopore sequencing, of the evaluated methods. This is in line with the data obtained by the team of Cuscó [23] who observed an increase of the fragment length when using Method ZR-HMW, in comparison with an alternative method using bead-beating and spin-column. As a result, these longer fragments helped the authors to generate longer contigs and improved the assembly of metagenomes from canine feces DNA sequenced by Nanopore [23]. In the present study, the DNA extraction methods were evaluated using R9.4.1 flowcells and 109 chemistry, which were the most stable and reliable at the time of the experiments. However, the Nanopore technology is rapidly evolving and the most advanced Nanopore technology includes now R10.4.1 flowcells with 114 chemistry, allowing a substantial improvement in read accuracy up to 99% and promising ultra-long read sequencing with N50 values above 50 kb. Considering these continuous improvements of the Nanopore technology with constantly increasing sequencing quality, it is definitely important to use a performant and reliable DNA extraction method, such as Method ZR-HMW, that can deliver pure HMW DNA to maximize the sequencing performance.

The use of enzymatic digestion, phenol–chloroform and gravity column, as performed with Method Mo, is recommended in the literature to obtain ultra-long DNA fragments for metagenomics studies [20, 21, 30]. However, this parameter could not be measured in the present study because the DNA yield obtained with our samples was too low when using this method. The range of concentrations used in our *B. subtilis* and *E.* *coli* experiments, although selected to be representative of the level of pathogen contaminations that can be present in a sample, might have been too low and too challenging for the tested methods, and could explain some of the low DNA yields obtained, i.e. for methods ZR and Mo. We applied the Method Mo as originally described by the team of Moss [20]. Nevertheless, the digestion and gravity column purification steps were omitted by Moss et al. when using a microbial community standard composed of 2.4 × 10^8^ cells (an amount higher than the one tested in our bacterial mixes), due to the limited concentration in DNA available [20]. Additionally, when testing the method on real stool samples, three samples were combined in one gravity column. These adaptations were probably needed to concentrate the DNA and increase the low yield of the method. Considering this, the bacterial mixes used in our study were perhaps not concentrated enough and not adapted to this method without specific modifications. Despite the fact that Method Mo was not selected for Nanopore sequencing with MCSII samples, it was decided to include the MetaPolyzyme from this method, in Method ZR-HMW, as replacement of lysozyme. Indeed, as MetaPolyzyme is a cocktail of enzymes designed for the lysis of a wide variety of microorganisms for microbiome studies [40], it is more adapted to deal with complex metagenomics samples. The incorporation of the MetaPolyzyme in the user-friendly protocol of Method ZR-HMW, which is less complex, time-consuming and without the use of hazardous chemical products, in comparison with Method Mo, gave excellent results in this study and was therefore a good combination.

Although obtaining good performance in this study, the Method ZR-HMW still requires various equipment, which impairs its use outside the laboratory in combination with the portable Nanopore technology. In contrast, the portable Method CB is composed of an all-included ingenious system of bead-beating tube connected to a battery for cell lysis, and luer-lock column compatible with syringes for sample handling during DNA purification. Nevertheless, the Nanopore sequencing experiments with MCSII samples showed that Method CB was less performant for obtaining a correct representation of the microbiome, especially in the presence of a complex matrix such as stool. To our knowledge, this is the first time that Method CB was evaluated and compared to other DNA extraction methods in the context of metagenomics applications using Nanopore sequencing. The data obtained in the present study shows that although the Nanopore platform is a tailored sequencing technology, allowing sequencing in isolated area as far as the International Space Station [41, 42] or high Arctic [43], the DNA extraction and purification techniques required for shotgun metagenomics with complex samples, are not ready yet to follow this level of portability. However, the best of methods ZR-HMW and CB could be merged to combine performance and portability, with the aim to offer a portable method adapted to an on-site sequencing workflow with Nanopore technology.

## Conclusions

In this study, several DNA extraction methods were evaluated for best compatibility with bacterial metagenomics using Nanopore sequencing. Considering all the results of the evaluated methods, Method ZR-HMW obtained the best performance with production of a high yield of HMW pure DNA, with a good sequencing depth, even for a complex sample such as a synthetic stool mix containing a lot of eukaryotic DNA. In future work, the flexibility of this method should be tested on a wide variety of metagenomics real samples, evaluating also the correct detection of viruses and eukaryotes, to test its performance for different case studies.

### Supplementary Information

**Additional file 1**. Supplementary Material 1

**Additional file 2. **Supplementary Material 2

**Additional file 3.** Supplementary Material 3

**Additional file 4.** Supplementary Material 4

## Data Availability

All data generated or analysed during this study are included in this published article and its supplementary information files. The dataset supporting the conclusions of this article is available in the NCBI Sequence Read Archive (SRA) repository, under the BioProject ID PRJNA954551: https://dataview.ncbi.nlm.nih.gov/object/PRJNA954551.

## References

[1] Hu T, Chitnis N, Monos D, Dinh A (2021). Next-generation sequencing technologies: An overview. Hum Immunol.

[2] Kwong JC, McCallum N, Sintchenko V, Howden BP (2015). Whole genome sequencing in clinical and public health microbiology. Pathology (Phila).

[3] Ellington MJ, Ekelund O, Aarestrup FM, Canton R, Doumith M, Giske C (2017). The role of whole genome sequencing in antimicrobial susceptibility testing of bacteria: report from the EUCAST Subcommittee. Clin Microbiol Infect Off Publ Eur Soc Clin Microbiol Infect Dis.

[4] Nouws S, Bogaerts B, Verhaegen B, Denayer S, Crombé F, De Rauw K (2020). The Benefits of Whole Genome Sequencing for Foodborne Outbreak Investigation from the Perspective of a National Reference Laboratory in a Smaller Country. Foods.

[5] Nouws S, Bogaerts B, Verhaegen B, Denayer S, Laeremans L, Marchal K, et al. Whole Genome Sequencing Provides an Added Value to the Investigation of Staphylococcal Food Poisoning Outbreaks. Front Microbiol. 2021;12:750278. 10.3389/fmicb.2021.750278 PMC8593433 34795649

[6] Su M, Satola SW, Read TD (2019). Genome-Based Prediction of Bacterial Antibiotic Resistance. J Clin Microbiol.

[7] Akaçin İ, Ersoy Ş, Doluca O, Güngörmüşler M (2022). Comparing the significance of the utilization of next generation and third generation sequencing technologies in microbial metagenomics. Microbiol Res.

[8] Carleton HA, Besser J, Williams-Newkirk AJ, Huang A, Trees E, Gerner-Smidt P (2019). Metagenomic Approaches for Public Health Surveillance of Foodborne Infections: Opportunities and Challenges. Foodborne Pathog Dis.

[9] Li N, Cai Q, Miao Q, Song Z, Fang Y, Hu B (2021). High-Throughput Metagenomics for Identification of Pathogens in the Clinical Settings. Small Methods.

[10] Ye SH, Siddle KJ, Park DJ, Sabeti PC (2019). Benchmarking Metagenomics Tools for Taxonomic Classification. Cell.

[11] Buytaers FE, Saltykova A, Denayer S, Verhaegen B, Vanneste K, Roosens NHC, et al. Towards Real-Time and Affordable Strain-Level Metagenomics-Based Foodborne Outbreak Investigations Using Oxford Nanopore Sequencing Technologies. Front Microbiol. 2021;12:738284. 10.3389/fmicb.2021.738284 PMC8602914 34803953

[12] Buytaers FE, Saltykova A, Mattheus W, Verhaegen B, Roosens NHC, Vanneste K (2021). Application of a strain-level shotgun metagenomics approach on food samples: resolution of the source of a Salmonella food-borne outbreak. Microb Genomics.

[13] Loman NJ, Constantinidou C, Christner M, Rohde H, Chan JZM, Quick J (2013). A culture-independent sequence-based metagenomics approach to the investigation of an outbreak of Shiga-toxigenic Escherichia coli O104:H4. JAMA.

[14] Ko KKK, Chng KR, Nagarajan N (2022). Metagenomics-enabled microbial surveillance Nat Microbiol.

[15] Berbers B, Saltykova A, Garcia-Graells C, Philipp P, Arella F, Marchal K (2020). Combining short and long read sequencing to characterize antimicrobial resistance genes on plasmids applied to an unauthorized genetically modified Bacillus. Sci Rep.

[16] D’aes J, Fraiture M-A, Bogaerts B, De Keersmaecker SCJ, Roosens NHC, Vanneste K (2021). Characterization of Genetically Modified Microorganisms Using Short- and Long-Read Whole-Genome Sequencing Reveals Contaminations of Related Origin in Multiple Commercial Food Enzyme Products. Foods.

[17] Leggett RM, Clark MD (2017). A world of opportunities with nanopore sequencing. J Exp Bot.

[18] Lamb HJ, Hayes BJ, Nguyen LT, Ross EM (2020). The Future of Livestock Management: A Review of Real-Time Portable Sequencing Applied to Livestock. Genes.

[19] Gardy JL, Loman NJ (2018). Towards a genomics-informed, real-time, global pathogen surveillance system. Nat Rev Genet.

[20] Moss EL, Maghini DG, Bhatt AS (2020). Complete, closed bacterial genomes from microbiomes using nanopore sequencing. Nat Biotechnol.

[21] Maghini DG, Moss EL, Vance SE, Bhatt AS (2021). Improved high-molecular-weight DNA extraction, nanopore sequencing and metagenomic assembly from the human gut microbiome. Nat Protoc.

[22] Sambrook J, Fritsch EF, Maniatis T (1989). Molecular Cloning: A Laboratory Manual.

[23] Cuscó A, Pérez D, Viñes J, Fàbregas N, Francino O (2021). Long-read metagenomics retrieves complete single-contig bacterial genomes from canine feces. BMC Genomics.

[24] Costea PI, Zeller G, Sunagawa S, Pelletier E, Alberti A, Levenez F (2017). Towards standards for human fecal sample processing in metagenomic studies. Nat Biotechnol.

[25] Walden C, Carbonero F, Zhang W (2017). Assessing impacts of DNA extraction methods on next generation sequencing of water and wastewater samples. J Microbiol Methods.

[26] Ducarmon QR, Hornung BVH, Geelen AR, Kuijper EJ, Zwittink RD (2020). Toward Standards in Clinical Microbiota Studies: Comparison of Three DNA Extraction Methods and Two Bioinformatic Pipelines. mSystems..

[27] Angelakis E, Bachar D, Henrissat B, Armougom F, Audoly G, Lagier J-C (2016). Glycans affect DNA extraction and induce substantial differences in gut metagenomic studies. Sci Rep.

[28] Hart ML, Meyer A, Johnson PJ, Ericsson AC (2015). Comparative Evaluation of DNA Extraction Methods from Feces of Multiple Host Species for Downstream Next-Generation Sequencing. PLoS ONE.

[29] Rehner J, Schmartz GP, Groeger L, Dastbaz J, Ludwig N, Hannig M (2022). Systematic Cross-biospecimen Evaluation of DNA Extraction Kits for Long- and Short-read Multi-metagenomic Sequencing Studies. Genomics Proteomics Bioinformatics.

[30] Trigodet F, Lolans K, Fogarty E, Shaiber A, Morrison HG, Barreiro L (2022). High molecular weight DNA extraction strategies for long-read sequencing of complex metagenomes. Mol Ecol Resour.

[31] Nicholls SM, Quick JC, Tang S, Loman NJ (2019). Ultra-deep, long-read nanopore sequencing of mock microbial community standards. GigaScience..

[32] Barbau-piednoir E, De Keersmaecker SCJ, Delvoye M, Gau C, Philipp P, Roosens NH (2015). Use of next generation sequencing data to develop a qPCR method for specific detection of EU-unauthorized genetically modified Bacillus subtilis overproducing riboflavin. BMC Biotechnol.

[33] Barbau-Piednoir E, De Keersmaecker SCJ, Wuyts V, Gau C, Pirovano W, Costessi A (2015). Genome Sequence of EU-Unauthorized Genetically Modified Bacillus subtilis Strain 2014–3557 Overproducing Riboflavin, Isolated from a Vitamin B2 80% Feed Additive. Genome Announc.

[34] Barbau-Piednoir E, Denayer S, Botteldoorn N, Dierick K, De Keersmaecker SCJ, Roosens NH (2018). Detection and discrimination of five E. coli pathotypes using a combinatory SYBR® Green qPCR screening system. Appl Microbiol Biotechnol..

[35] De Coster W, D’Hert S, Schultz DT, Cruts M, Van Broeckhoven C (2018). NanoPack: visualizing and processing long-read sequencing data. Bioinforma Oxf Engl.

[36] Wood DE, Salzberg SL (2014). Kraken: ultrafast metagenomic sequence classification using exact alignments. Genome Biol.

[37] Wood DE, Lu J, Langmead B (2019). Improved metagenomic analysis with Kraken 2. Genome Biol.

[38] Portik DM, Brown CT, Pierce-Ward NT (2022). Evaluation of taxonomic classification and profiling methods for long-read shotgun metagenomic sequencing datasets. BMC Bioinformatics.

[39] Sun Z, Zhu D (2019). Exposure to outdoor air pollution and its human health outcomes: A scoping review. PLoS ONE.

[40] Tighe S, Afshinnekoo E, Rock TM, McGrath K, Alexander N, McIntyre A (2017). Genomic Methods and Microbiological Technologies for Profiling Novel and Extreme Environments for the Extreme Microbiome Project (XMP). J Biomol Tech JBT.

[41] Castro-Wallace SL, Chiu CY, John KK, Stahl SE, Rubins KH, McIntyre ABR (2017). Nanopore DNA Sequencing and Genome Assembly on the International Space Station. Sci Rep.

[42] Stahl-Rommel S, Jain M, Nguyen HN, Arnold RR, Aunon-Chancellor SM, Sharp GM (2021). Real-Time Culture-Independent Microbial Profiling Onboard the International Space Station Using Nanopore Sequencing. Genes.

[43] Goordial J, Altshuler I, Hindson K, Chan-Yam K, Marcolefas E, Whyte LG. In Situ Field Sequencing and Life Detection in Remote (79°26′N) Canadian High Arctic Permafrost Ice Wedge Microbial Communities. Front Microbiol. 2017;8:2594. 10.3389/fmicb.2017.02594 PMC5742409 29326684

